# Associations between canine temperament and salivary concentrations of cortisol and serotonin

**DOI:** 10.1371/journal.pone.0337781

**Published:** 2026-02-04

**Authors:** Youngwook Jung, Yujin Song, Kayoung Yang, Kyongwon Yoo, Youngtae Heo, Minjung Yoon

**Affiliations:** 1 Department of Animal Science and Biotechnology, Kyungpook National University, Sangju, Republic of Korea; 2 Animal Welfare Division, National Institute of Animal Science, Wanju, Republic of Korea; 3 Department of Companion Animal, Osan University, Osan, Republic of Korea; 4 Department of Horse, Companion and Wild Animal Science, Kyungpook National University, Sangju, Republic of Korea; 5 Research Institute for Innovative Animal Science, Kyungpook National University, Sangju, Republic of Korea; University of Messina, ITALY

## Abstract

Temperament influences canine behavior and helps determine a dog’s suitability as a companion or working animal. Although several temperament assessments exist, many rely on subjective evaluations, highlighting the need for scientifically validated methods. This study evaluated the validity of the Wesen temperament test by examining its association with physiological biomarkers—salivary cortisol and serotonin. Twenty-four dogs (11 females and 13 males) completed a modified Wesen test comprising seven subtests: Unconscious Confidence, Sociality, Noise Stability, Movement Stability, Desire for Play and Predation, Behavior in Stressful Situations, and Separation. Saliva samples were collected pre- and post-assessment, and cortisol and serotonin concentrations were measured using ELISA. Hormonal concentrations and temperament scores were analyzed using correlation, regression, and Kruskal-Wallis tests. Pre-assessment cortisol concentrations were negatively correlated with total average temperament score and several subtest scores. Post-assessment cortisol concentrations showed significant negative correlations with total average scores and all subtests. Changes in cortisol concentrations from pre- to post-assessment were negatively associated with temperament scores. Dogs with higher temperament scores exhibited significantly higher serotonin concentrations than those with lower scores. These results support the utility of temperament assessments validated through physiological markers and provide novel evidence linking canine temperament with endocrine function.

## Introduction

The temperament of dogs is a key factor in determining their suitability as companion animals [1]. Additionally, temperament plays a critical role in working contexts, such as guiding [2], detection [3], and rescue operations [4]. Therefore, selecting dogs with appropriate temperaments for specific purposes is important. Temperament assessment can help predict a dog’s future performance and suitability [5]. Several tools have been developed for this purpose, including the Canine Behaviour Assessment and Research Questionnaire (C-BARQ) [6], the In-For Training test [7], and the Wesen test [8]. These assessment tools differ in their evaluation methods: some rely on owners’ reports, while others require trained professionals. This variability raises concerns about potential subjectivity [7]. Importantly, temperament is not expressed uniformly across situations but can vary depending on environmental context and task demands. Previous studies have shown that dogs may exhibit different behavioral responses when exposed to distinct testing environments or challenges [9–11]. Given this context-dependent nature of temperament expression, it is necessary to validate temperament assessments using objective parameters. Thus, several studies have examined the validity of temperament assessments by comparing their scores with physiological biomarkers such as heart rate, respiratory rate, and hormone concentrations [12–14]. Among these biomarkers, hormones have emerged as indicators owing to their close association with temperament and behavior.

Hormonal responses are regulated by the neuroendocrine system. In particular, the hypothalamic–pituitary–adrenal (HPA) axis plays a central role in managing stress and maintaining homeostasis under stressful conditions [15–18]. HPA axis activity is coordinated through positive and negative feedback loops that regulate the production of key hormones, including cortisol.

As a key hormone of the HPA axis, cortisol is secreted in response to stress or negative emotional states [19]. It is commonly used as a biomarker of stress and overall well-being in dogs. Recent research has focused on the relationship between cortisol and temperament. Rosado and colleagues reported higher cortisol concentrations in aggressive dogs than in non-aggressive individuals [20]. Basal cortisol concentrations have also been linked to fear-related behaviors, such as panting and tongue protrusion [21]. Additionally, cortisol concentrations vary with the degree of sociality [22] and have been associated with touch sensitivity [23] and anxiety towards strangers [24]. Collectively, this evidence indicates that cortisol is involved in several aspects of canine temperament, including aggression, fear responses, and social interaction. Therefore, cortisol serves as an indicator of temperament-related traits beyond its traditional role in stress response.

Cortisol has been measured in several biological matrices, including plasma, urine, feces, hair, feathers, and saliva [21,25–27]. Blood cortisol is considered a reliable indicator of acute stress responses. However, because blood sampling is invasive, it has become less frequently used in recent studies. In contrast, saliva sampling has gained popularity as a noninvasive method. Steroid hormones can diffuse rapidly into saliva from the bloodstream owing to their molecular properties [28]. Moreover, salivary cortisol reliably reflects plasma cortisol concentrations in dogs [29]. Therefore, we selected saliva sampling as the method for cortisol measurement.

Serotonin plays a well-established role in temperament and behavior, including aggression, anxiety, and social interactions. Several studies have reported substantial differences in serotonin concentrations between aggressive and non-aggressive dogs [20,30–32]. Vermeire and colleagues further reported that serotonin receptors are expressed in cortical regions of the brain and are associated with anxiety disorders in dogs [33]. Associations between serotonin concentrations and dogs’ social behaviors toward humans have also been observed [34]. Collectively, these findings suggest that serotonin is a biomarker for temperament assessment in dogs.

In dogs, serotonin has predominantly been measured in blood samples [32,35–37]. However, the invasiveness of blood sampling may limit its use in temperament monitoring. Saliva sampling has therefore gained increasing attention as a relatively noninvasive approach. In humans, although salivary serotonin is not directly correlated with serotonin concentrations in cerebrospinal fluid or platelets, it provides a reliable measure of peripheral serotonin concentrations [38,39]. Furthermore, salivary serotonin concentrations have been associated with indicators of happiness, mood, and depressive disorders [40–42]. On this basis, saliva sampling was adopted as the method for measuring serotonin concentrations in this study.

This study aimed to validate a temperament assessment tool by measuring salivary cortisol and serotonin concentrations. Specifically, we (i) examined correlations between temperament assessment scores and cortisol and serotonin hormone concentrations and (ii) compared hormone concentrations between high- and low-scoring groups. The Wesen test, which was selected for temperament assessment in this study, was originally developed for German Shepherd dogs aged 9–13 months to evaluate behavioral responses across multiple domains [43]. However, its effectiveness and adaptability have led to its broad application across breeds and age groups [44]. We hypothesized that salivary hormone concentrations would correlate with temperament traits assessed by the Wesen test, thereby providing physiological evidence for the convergent validity of the test. The observations of this study may provide a scientific basis for the use of temperament assessments in dogs.

## Materials and methods

### Subjects

Twenty-four dogs (eleven females and thirteen males) participated in the study. The study included seven mixed-breed dogs, six Beagles, four Border Collies, two Labrador Retrievers, two Malinois, one Pomeranian, one French Bulldog, and one Shepherd. The mean age was 5.125 ± 3.059 years. The Beagles were bred for experimental use, whereas the remaining dogs were owned by private individuals or professional trainers. All dogs were routinely managed under veterinary care. No additional clinical procedures or medications were administered before or during the temperament assessment. In addition, none of the dogs had prior experience with temperament assessment. All experimental procedures were conducted in accordance with institutional ethical guidelines and were approved by the Animal Experimentation Ethics Committee of Kyungpook National University (approval number: 2024−0531).

### Temperament assessment

**Fig 1:**
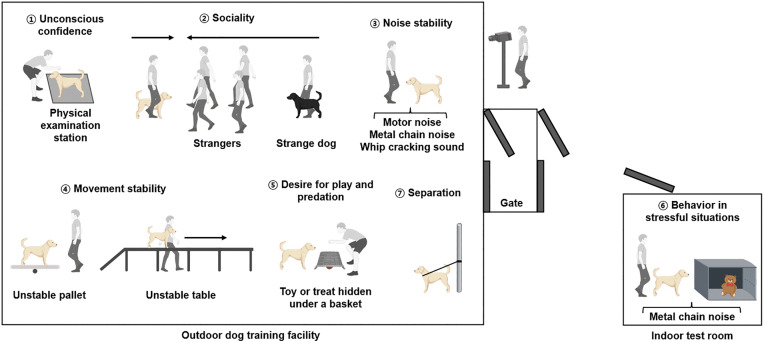
Schematic representation of the temperament assessment test process (created using BioRender.com).

To minimize stress associated with transportation, temperament assessments were conducted at three locations near the dogs’ original environments. All locations were arranged to provide comparable environmental conditions and were equipped with functionally equivalent testing equipment to ensure procedural consistency. The assessment was conducted by a single experienced international dog show judge. In this study, we applied a modified version of the Wesen test (Wesensüberprüfung). An overview of the test process is presented in Fig 1. Ruefenacht and colleagues noted that, in the environmental stimulus component of the Wesen test, the specific methods and objects used may vary depending on testing conditions [5]. In the present study, the core structure and evaluation principles of the original Wesen test were retained. Minor procedural adjustments were implemented only where necessary to address practical and safety-related constraints associated with the testing environment, without altering the conceptual framework or scoring criteria of the assessment. The modified version used in this study consisted of seven subtests [5,8,43]:

1. Unconscious Confidence: The judge conducted a physical examination while the handler kept the dog on a leash at a designated station. The examination included inspection of the teeth, testes (in males), and a full-body check. During this procedure, the dog’s attention, fear, confidence, interest, and relaxation were assessed.
2. Sociality: Four to six unfamiliar people formed a line and walked past the dog and the handler. Subsequently, a stranger handling an unfamiliar dog approached the tested dog and handler. During these encounters, responsiveness to the handler’s cues, confidence, cheerfulness, affinity, and energy were assessed.
3. Noise Stability: The dog was exposed to a series of auditory stimuli produced by a motor, a metal chain, and a whip. During exposure to these sounds, the dog’s calmness, relaxation, fear, and confidence were assessed.
4. Movement Stability: Dogs were guided by their handlers to sit and walk on an unstable pallet. They were then guided to ascend a series of connected tables via a ramp and to walk across them. During these tasks, relaxation, stability, fear, and activity were evaluated.
5. Desire for Play and Predation: The handler first played with the dog using a toy or treat. A stranger then interacted with the dog using the same object. Subsequently, the dog was encouraged to search for the object after it had been hidden under a box. During this subtest, activity, persistence, affinity, and interest were evaluated.
6. Behavior in Stressful Situations: This subtest was conducted in an indoor test room. The dog first played with a toy provided by the handler. The toy was then hidden to encourage a search. During the search, a sudden loud noise was produced by dropping a metal chain to simulate a stress-inducing stimulus. During this subtest, activity, confidence, persistence, interest, and reactions to the noise were evaluated.
7. Separation: Dogs were secured to a fixed object and left alone for 5 min. After this period, the dog encountered a stranger. During this subtest, anxiety, relaxation, and affinity were evaluated.

Each subtest comprised two to four categories, scored on a five-point scale (1 = lowest; 5 = highest). The judge evaluated all reactions according to standardized guidelines. The entire temperament assessment was video-recorded by a designated observer. All subtests were conducted at an outdoor dog-training facility, except for the Behavior in Stressful Situations subtest, which took place in an indoor test room.

### Sample collection

To analyze hormone concentrations, saliva samples were collected from each dog before and after temperament assessment by an experimenter who was not involved in judging. To account for circadian variation, all sample collections were conducted during a similar time period in the morning. Samples were collected using a swab device and storage tube (Salimetrics, Carlsbad, CA, USA) by gently placing the swab under the tongue and in the cheek pouch. During transport to the laboratory, the samples were kept in an icebox at 4 °C to maintain integrity. Upon arrival, saliva was centrifuged at 1,000 × *g* for 2 min at 4 °C and stored at −80 °C until analysis.

### Hormone analysis

Hormone concentrations were measured using commercial enzyme-linked immunosorbent assay (ELISA) kits. Samples were analyzed in duplicate without prior extraction or dilution. Optical density was measured using a microplate reader (Tecan, Männedorf, Switzerland), and concentrations were calculated based on a four-parameter logistic standard curve.

#### Cortisol ELISA.

Salivary cortisol concentrations before and after temperament assessment were measured using a Cortisol ELISA kit (1–3002; Salimetrics) with a sensitivity of 0.018 μg/dL. The intra- and inter-assay coefficients of variation (CVs) were 1.554% and 3.572%, respectively. Optical density was measured at 450 nm following the manufacturer’s instructions.

#### Serotonin ELISA.

Owing to insufficient saliva volume, sixteen samples that met the minimum quantity required for ELISA were included in the serotonin analysis. Salivary serotonin concentrations before temperament assessment were determined using a Serotonin ELISA kit (ADI-900–175; Enzo Life Sciences, Farmingdale, NY, USA) with a sensitivity of 0.293 ng/mL. The intra- and inter-assay CVs were 7.080% and 14.970%, respectively. Optical density was measured at 405 nm following the manufacturer’s protocol.

### Statistical analysis

Statistical analyses were performed using IBM SPSS Statistics (version 27; IBM, Armonk, NY, USA), and data visualization was conducted using GraphPad Prism (version 9; GraphPad Software, San Diego, CA, USA). Data normality was assessed using the Shapiro–Wilk test. Sex differences in hormone concentrations were evaluated using the Mann–Whitney *U* test. Variations in hormone concentrations among dogs bred for experimental use, privately owned dogs, and dogs raised by professional trainers were evaluated using the Kruskal–Wallis test. Spearman’s rank correlation coefficient (ρ) was used to examine relationships between hormone concentrations and temperament scores. Additionally, both Spearman’s ρ and linear regression analyses were used to evaluate associations between changes in cortisol concentrations and temperament assessment scores. The Kruskal–Wallis test was also used to compare hormone concentrations across groups categorized by temperament assessment scores. Hormone concentrations and temperament assessment scores are presented as mean ± standard error of the mean. Statistical significance was set at *p* < 0.050, and values between 0.050 and 0.100 were considered indicative of a trend.

## Results

### Correlation between cortisol concentrations and temperament assessment scores

To examine potential sex effects, hormone concentrations were compared between females and males. No significant differences in cortisol or serotonin concentrations were observed between sexes or among dog populations. Therefore, data were pooled across sex and population for subsequent analyses. Correlations between salivary cortisol concentrations and temperament assessment scores are presented in Table 1. Pre-assessment cortisol concentrations were negatively correlated with total average scores (*p* = 0.036). Negative correlations were also observed between pre-assessment cortisol concentrations and several subtests, including Unconscious Confidence (*p* = 0.006), Sociality (*p* = 0.035), Noise Stability (*p* = 0.003), and Separation (*p* = 0.006). No significant correlations were observed between pre-assessment cortisol concentrations and other subtests, including Movement Stability (*p* = 0.291), Desire for Play and Predation (*p* = 0.801), and Behavior in Stressful Situations (*p* = 0.126). Post-assessment cortisol concentrations were negatively correlated with all subtest scores as well as with the total average scores (*p* < 0.001).

**Table 1:** Correlations between temperament assessment scores and salivary cortisol and serotonin concentrations.

		Total average scores	Unconscious confidence	Sociality	Noise stability	Movement stability	Desire for play and predation	Behavior in stressful situations	Separation
Cortisol (Pre)	Spearman’s ρ	−0.430*	−0.544**	−0.432*	−0.586**	−0.225	−0.054	−0.321	−0.540**
	*p*-value	0.036	0.006	0.035	0.003	0.291	0.801	0.126	0.006
Cortisol (Post)	Spearman’s ρ	−0.714**	−0.540**	−0.557**	−0.705**	−0.617**	−0.497*	−0.598**	−0.817**
	*p*-value	<0.001	0.006	0.005	<0.001	0.001	0.013	0.002	<0.001
Serotonin	Spearman’s ρ	0.474^**†**^	0.396	0.406	0.364	0.633**	0.417	0.289	0.369
	*p*-value	0.064	0.129	0.118	0.165	0.009	0.108	0.277	0.159

** *p* < 0.010, * *p* < 0.050, † 0.050 < *p* < 0.100

Pre and Post indicate samples collected before and after temperament assessment, respectively.

### Correlation between changes in cortisol concentrations and temperament assessment scores

**Fig 2:**
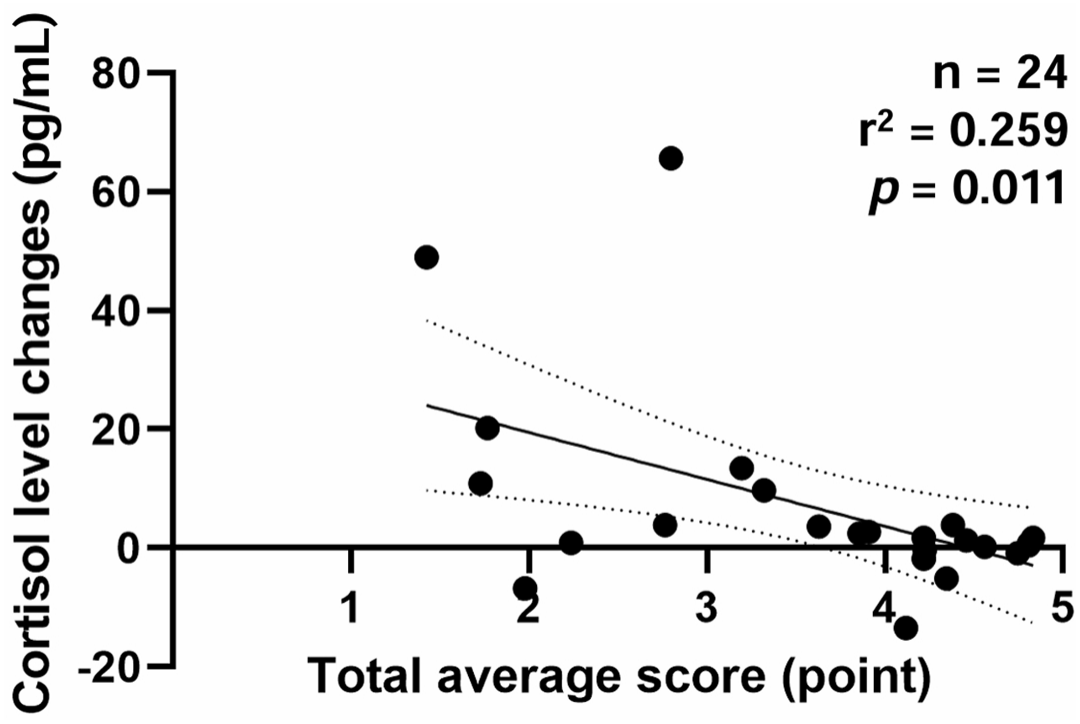
Linear regression between changes in cortisol concentrations (pre- to post-assessment) and total average temperament assessment scores. The analysis indicates a negative correlation (Spearman’s ρ = −0.526, *p* = 0.008) with an *r*² of 0.259 (*p* = 0.011) and an adjusted *r*² of 0.225.

The correlation between changes in cortisol concentrations (from pre- to post-assessment) and total average scores is illustrated in Fig 2. Cortisol concentrations were negatively correlated with total average scores (*p* = 0.008). The coefficient of determination (*r*²) was 0.259 (*p* = 0.011), and the adjusted *r*² was 0.225.

### Comparison of cortisol concentrations among three groups

**Fig 3:**
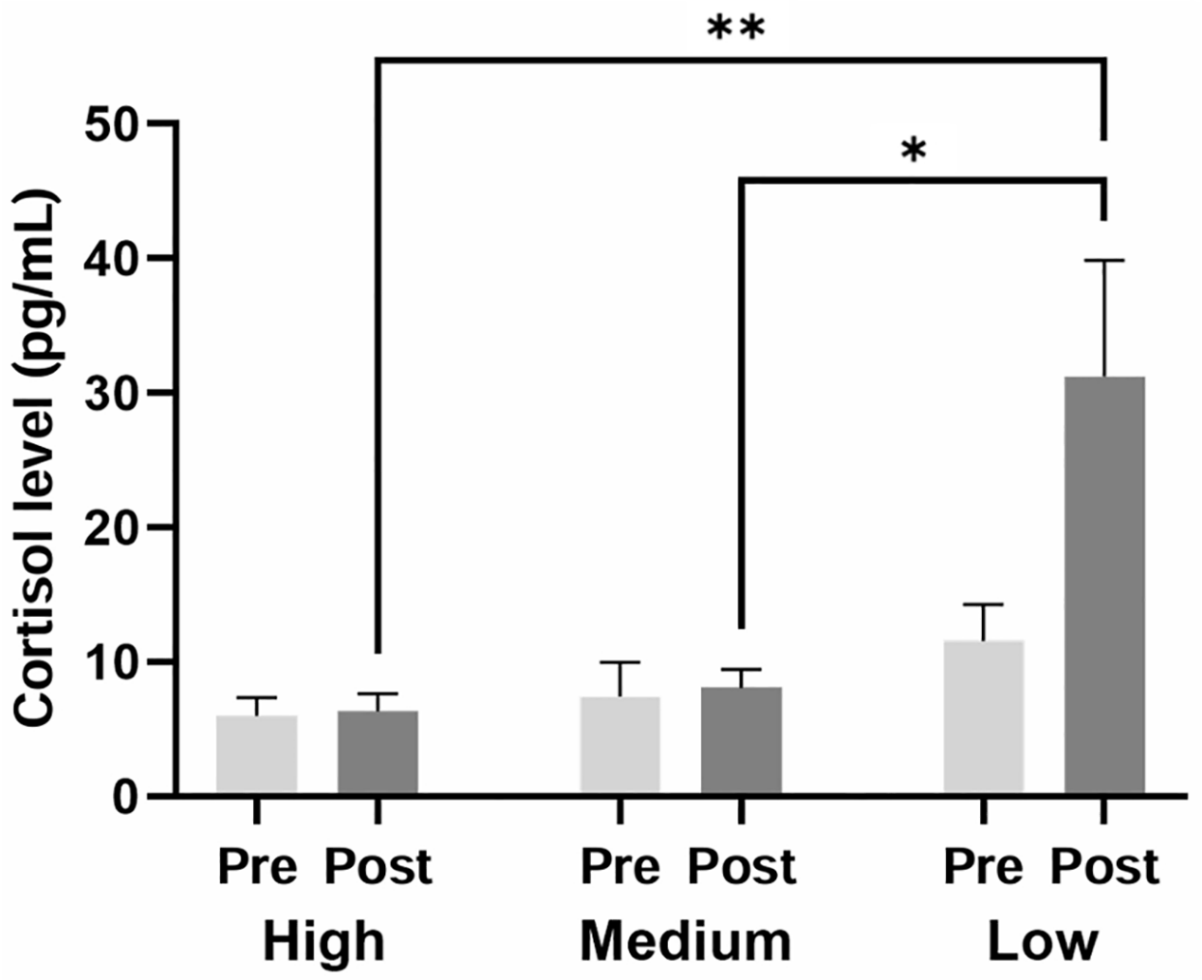
Salivary cortisol concentrations in high-, medium-, and low-scoring groups. Cortisol concentrations were 5.996 ± 1.258 pg/mL (pre) and 6.295 ± 1.283 pg/mL (post) in the high-scoring group, 7.441 ± 2.379 pg/mL (pre) and 8.044 ± 1.296 pg/mL (post) in the medium-scoring group, and 11.581 ± 2.535 pg/mL (pre) and 31.213 ± 8.075 pg/mL (post) in the low-scoring group. Pre and Post indicate samples collected before and after temperament assessment, respectively. ** *p* < 0.010, * *p* < 0.050.

Cortisol concentrations tended to increase from pre- to post-assessment (8.339 ± 1.352 pg/mL vs. 15.184 ± 3.682 pg/mL; *p* = 0.062). To further investigate these differences, dogs were categorized into three scoring groups—high (n = 8), medium (n = 8), and low (n = 8)—based on their total average temperament assessment scores (Table 2). These groups differed significantly in their total average scores (*p* < 0.010). Pre-assessment cortisol concentrations tended to be lower in the high-scoring group compared with the low-scoring group (*p* = 0.058; Fig 3). Post-assessment cortisol concentrations were higher in the low-scoring group than in both the high-scoring (*p* = 0.005) and medium-scoring (*p* = 0.012) groups. Within the low-scoring group, post-assessment cortisol concentrations also tended to be higher than pre-assessment concentrations (*p* = 0.059).

**Table 2:** Average temperament assessment scores of each group for cortisol and serotonin analyses.

	High	Medium	Low
Cortisol	4.618 ± 0.068^a^	3.937 ± 0.109^b^	2.237 ± 0.206^c^
Serotonin	4.368 ± 0.093^a^	3.360 ± 0.163^b^	1.828 ± 0.121^c^

Different superscripts indicate a significant difference (*p* < 0.010).

### Correlation between serotonin concentrations and temperament assessment scores

Correlations between salivary serotonin concentrations and temperament assessment scores are summarized in Table 1. A trend toward a positive correlation was observed between serotonin concentrations and the total average scores, although this result was not statistically significant (*p* = 0.064). In contrast, a significant positive correlation was identified between serotonin concentrations and the Movement Stability subtest (*p* = 0.009). No significant correlations were observed for the remaining subtests, including Unconscious Confidence (*p* = 0.129), Sociality (*p* = 0.118), Noise Stability (*p* = 0.165), Desire for Play and Predation (*p* = 0.108), Behavior in Stressful Situations (*p* = 0.277), and Separation (*p* = 0.159).

### Comparison of serotonin concentrations among three groups

**Fig 4:**
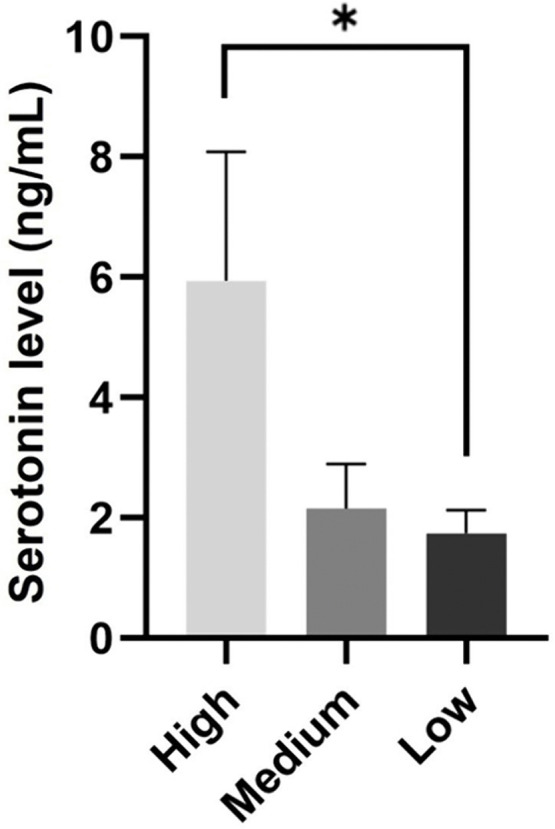
Salivary serotonin concentrations in high-, medium-, and low-scoring groups. Serotonin concentrations were 5.943 ± 1.958 ng/mL in the high-scoring group, 2.154 ± 0.666 ng/mL in the medium-scoring group, and 1.742 ± 0.348 ng/mL in the low-scoring group. * *p* < 0.050.

To compare serotonin concentrations among groups, dogs were categorized into high- (n = 6), medium- (n = 5), and low-scoring (n = 5) groups based on their total average temperament assessment scores (Table 2). Significant differences in the total average scores were observed among the groups (*p* < 0.010). Serotonin concentrations in the high-scoring group were significantly higher than those in the low-scoring group (*p* = 0.028; Fig 4).

## Discussion

In this study, we validated a temperament assessment based on the Wesen test by incorporating hormonal markers as physiological indicators. To our knowledge, this is the first study to physiologically validate the Wesen test using noninvasive salivary cortisol and serotonin measurements in dogs. This approach allows behavioral assessment outcomes to be interpreted in relation to objective endocrine markers. Establishing such physiological evidence strengthens the foundation for accurately evaluating canine temperament. A reliable assessment tool can help determine a dog’s suitability as a companion animal or for specific working purposes. Moreover, physiologically validated assessments may contribute to policies and legislation that promote the safe selection and responsible adoption of dogs with appropriate temperaments.

We examined associations between temperament assessment scores and salivary cortisol concentrations. Pre-assessment cortisol concentrations were negatively correlated with the total average score and several subtests. Post-assessment cortisol concentrations showed negative correlations with the total average score and all subtests. These observations provide physiological evidence supporting the convergent validity of the Wesen test. Lower cortisol concentrations, an established biomarker of acute stress and arousal in dogs, were associated with behavioral profiles suggestive of greater emotional stability. Our results align with previous studies reporting associations between cortisol concentrations and behavioral traits. Rayment and colleagues reported a slight positive correlation between cortisol concentrations and C-BARQ traits such as separation-related problems and touch sensitivity [23]. Similarly, McPeake and colleagues observed a negative correlation between post-assessment salivary cortisol concentrations and frustration-coping ability [13]. In a study investigating the relationship between long-term stress and temperament, Roth and colleagues examined associations between hair cortisol concentrations and canine temperament [12]. They found that hair cortisol concentrations were positively correlated with stranger-directed aggression and chasing traits, as assessed using the C-BARQ. Rossi and colleagues also reported that lower cortisol concentrations were associated with more frequent play-soliciting and longer durations of exploratory behavior [45]. Collectively, these outcomes suggest that cortisol concentrations reflect behavioral traits related to stress responsiveness and emotional regulation across several temperament assessments.

In this study, we identified a relationship between temperament assessment scores and changes in salivary cortisol concentrations. Dogs with higher temperament assessment scores exhibited more stable physiological responses. The negative correlation between cortisol changes and Wesen test scores suggests that behaviorally well-adjusted dogs may be more resilient to stress. From a biological perspective, cortisol is a primary hormone of the HPA axis, and changes in salivary cortisol reflect individual differences in HPA axis–mediated stress responsiveness [15,16]. These findings provide physiological support for the convergent validity of the Wesen test in assessing emotional stability and stress reactivity in dogs. Similar results have been reported in other studies. McPeake and colleagues reported significant correlations between scores on the Canine Frustration Questionnaire and changes in salivary cortisol concentrations [13]. Similarly, the startling test has been validated through its association with physiological indicators such as cortisol concentrations and heart rate [14]. Another study found that high sociability, as measured by behavioral assessment tests, was associated with smaller variations in cortisol concentrations [22]. Overall, this evidence highlights a recurring association between canine temperament traits, as assessed across several temperament tools, and physiological responses related to stress.

Additionally, we compared cortisol concentrations among three groups classified by temperament assessment scores. Pre-assessment cortisol concentrations tended to be lower in the high-scoring group than in the low-scoring group, consistent with reports that shelter dogs with more sociable temperaments exhibit lower baseline cortisol concentrations [46]. Likewise, dogs engaging in more positive interactions generally exhibit lower cortisol concentrations [47]. Post-assessment cortisol concentrations were significantly higher in the low-scoring group than in the medium- and high-scoring groups, indicating greater stress reactivity among dogs with lower temperament scores. Consistent with this finding, Shin and Shin reported that more sociable dogs exhibited lower cortisol concentrations following behavioral assessment than less sociable dogs [22]. These results suggest that dogs with more stable or socially adaptive temperaments are less reactive to testing situations and experience lower stress concentrations. In the low-scoring group, cortisol concentrations tended to increase after the assessment, reflecting the overall trend observed in this study. Collectively, these results suggest that both basal and reactive cortisol concentrations may be associated with certain aspects of canine temperament, particularly in relation to stress responsiveness. In this context, socially adaptive dogs tended to exhibit relatively lower physiological stress responses in both routine and test-related situations.

Interestingly, Lensen and colleagues measured salivary cortisol and chromogranin A (CgA) concentrations 10 min after a behavioral test in adult dogs [48]. They reported that salivary cortisol concentrations were positively associated with high trainability, whereas CgA concentrations were negatively associated with stranger-directed fear. However, these associations varied depending on the timing of sample collection. Cortisol concentrations measured 40 min after the test were associated with high energy, whereas CgA concentrations were related to low excitability, both of which may be considered undesirable traits. These findings highlight the importance of sampling time when interpreting stress-related biomarkers. In the present study, saliva samples were collected at only two time points (pre- and post-assessment), which limits the temporal resolution of endocrine responses and constrains interpretation of dynamic physiological changes. Future studies should incorporate multiple sampling points to better characterize hormonal response patterns, including peak responses and recovery phases.

We also analyzed the relationship between temperament assessment scores and salivary serotonin concentrations. Although overall temperament scores did not show a statistically significant correlation with serotonin concentrations, a positive trend was observed. This result suggests that behavioral indicators of emotional stability are partly associated with serotonergic activity. Notably, the Movement Stability subtest, which evaluates traits such as relaxation, stability, and activeness, showed a significant positive correlation with serotonin concentrations. This observation supports the convergent validity of the subtest, suggesting that higher serotonin concentrations may be associated with calmer and more regulated behavioral profiles. Serotonin has also been proposed as a marker of stress responsiveness. Previous studies across multiple species, including cattle, swine, dogs, and horses, have demonstrated that serotonin concentrations can change in response to stressful or clinical procedures and may serve as a physiological marker related to stress, pain, or oxidative processes [49–52]. These comparative findings support the relevance of serotonin as a stress biomarker. However, studies investigating acute stress responses have reported that serotonin-related measures vary depending on the timing of post-stressor sampling, which differs across study designs [53,54]. Accordingly, future studies should incorporate repeated sampling at multiple time points to better characterize serotonergic dynamics. In the present study, however, salivary serotonin was measured only at baseline. Therefore, the current findings should be interpreted with caution, as they do not capture dynamic serotonergic responses to the temperament assessment itself. Taken together, given the multifactorial role of serotonin, the present results likely reflect general associations with temperament-related behaviors rather than specific emotional or stress-related mechanisms [51].

These results are consistent with previous observations on serotonin–behavior relationships in dogs. Rayment and colleagues reported a negative trend between serotonin concentrations and C-BARQ traits related to separation-related problems and touch sensitivity [23]. Similarly, another study identified a weak linear correlation between social behavioral scores and serotonin concentrations in shelter dogs [34]. Wright and colleagues also found that higher impulsivity scores on the Dog Impulsivity Assessment Scale (DIAS) were associated with lower serotonin concentrations [55]. Taken together, these studies and our findings suggest that serotonin concentrations are associated with several positive behavioral characteristics across diverse behavioral assessment tools. This highlights the potential of serotonin as a physiological marker of emotional and behavioral regulation in dogs.

We also compared serotonin concentrations among three groups classified by temperament scores. Dogs in the high-scoring group exhibited significantly higher serotonin concentrations than those in the low-scoring group. This finding aligns with numerous studies reporting that higher serotonin concentrations are associated with lower aggression and anxiety in dogs [34,37,56–58]. Collectively, these observations provide physiological evidence supporting the reliability of the temperament assessment used in this study. However, one study reported no significant differences in serotonin concentrations between groups classified by factors assessed through the C-BARQ or DIAS [23]. This inconsistency may stem from methodological differences. Unlike the temperament assessment in our study, which involved direct behavioral observation, both the C-BARQ and DIAS rely on owner-reported data. Although these tools are validated, their outcomes can be influenced by individual variation in owner perception and experience. Moreover, previous studies suggest that factors such as learning history, environmental conditions, and breed characteristics also influence test outcomes [23]. Therefore, these factors should be considered when interpreting associations between behavioral assessments and physiological markers.

Although this study yielded several noteworthy findings, certain limitations should be considered when interpreting the results. The overall sample size limits statistical power and increases the possibility of both Type I and Type II errors. In particular, owing to constraints in salivary sampling, fewer samples were available for serotonin analysis, resulting in a smaller dataset compared with that of cortisol. To improve reliability and generalizability, future studies should include a larger number of dogs. Previous research has shown that salivary stimulants do not significantly affect hormonal measurements [59]. Thus, their use may be considered in future studies to increase saliva yield. In this study, no significant differences in hormone concentrations were observed between groups classified by sex or living environment. However, these findings should be interpreted with caution, as the limited sample size may have reduced the ability to detect group differences. Cobb and colleagues reported that variables such as breed and body weight have minimal effects on salivary cortisol concentrations [59]. However, they also found that intact females exhibited significantly higher cortisol concentrations than intact males and neutered dogs. Additionally, living environment influenced cortisol concentrations, and puppies under 6 months of age exhibited significantly lower cortisol concentrations than older dogs. Similarly, Sandri and colleagues found that cortisol concentrations were associated with body size and housing conditions [60]. Collectively, these findings indicate that biological factors—including body size, sex, reproductive status, and age—may influence basal hormonal concentrations. In addition, environmental factors such as housing conditions, prior handling, and diet may also contribute to variability in hormonal responses [23]. Future research should explore how these factors interact with temperament assessment to clarify their combined effects on physiological stress responses in dogs. Although cortisol is a well-established biomarker of stress, future studies should incorporate other physiological indicators to improve the robustness of results [61]. Heart rate is one such parameter. For example, Robinson and colleagues reported that heart rate correlated with several behavioral test batteries [62]. Moreover, King and colleagues found a strong correlation between heart rate and startling test scores [14]. Incorporating validated physiological markers and examining their interrelationships may enhance the reliability and robustness of future findings.

In conclusion, this study validated a canine temperament assessment by examining its associations with cortisol and serotonin. Significant correlations were observed between temperament assessment scores and cortisol concentrations, and a potential association was observed with serotonin concentrations. Furthermore, both hormonal markers differed among groups categorized by temperament scores. Taken together, these findings suggest that cortisol and serotonin may be associated with certain temperament-related aspects of stress reactivity and emotional regulation in dogs. At the same time, because these biomarkers are influenced by multiple physiological and psychological processes, further studies are warranted to more precisely clarify their roles. These results support the utility of temperament assessments validated through physiological measures and contribute to a better understanding of the relationships among canine temperament, behavior, and endocrine function. Future studies incorporating larger and more diverse populations and extending this physiological validation framework to other temperament assessment tools may further enhance its robustness and practical applicability.
